# The Novodiag^®^ Stool parasites assay, an innovative high-plex technique for fast detection of protozoa, helminths and microsporidia in stool samples: a retrospective and prospective study

**DOI:** 10.1051/parasite/2022026

**Published:** 2022-05-13

**Authors:** Sophie Hartuis, Rose-Anne Lavergne, Céline Nourrisson, Jaco Verweij, Guillaume Desoubeaux, Florian Lussac-Sorton, Jean-Philippe Lemoine, Estelle Cateau, Fakhri Jeddi, Philippe Poirier, Patrice Le Pape, Florent Morio

**Affiliations:** 1 Nantes Université, CHU de Nantes, Cibles et Médicaments des Infections et de l’Immunité, IICiMed, UR1155 44000 Nantes France; 2 Laboratoire de Parasitologie–Mycologie, Centre de Biologie, CHU Gabriel Montpied 58 rue Montalembert 63000 Clermont-Ferrand France; 3 Elisabeth-TweeSteden Hospital, Microvida Laboratory for Medical Microbiology and Immunology PO Box 90151 5000 Tilburg The Netherlands; 4 Service de Parasitologie – Mycologie – Médecine Tropicale, CHRU de Tours 2 Boulevard Tonnellé 37000 Tours France; 5 Laboratoire de Parasitologie–Mycologie PTMI – Groupe Hospitalier Pellegrin Place Amélie Raba Léon 33076 Bordeaux France; 6 Laboratoire de Parasitologie–Mycologie, CHU 4 rue Larrey 49033 Angers France; 7 Laboratoire de Parasitologie et Mycologie Médicales, CHU La Miletrie BP 577 86021 Poitiers Cedex France

**Keywords:** Stool, Gastrointestinal parasites, Microscopy, Novodiag^®^ Stool Parasites, High-plex detection, Prospective study, Comparative evaluation

## Abstract

*Objectives*: We provide the first evaluation of the CE-IVD marked Novodiag^®^ stool parasites assay (NVD), allowing rapid and high-plex detection of 26 distinct targets, encompassing protozoans, helminths and microsporidia in stool samples. *Methods*: A total of 254 samples (*n* = 205 patients) were prospectively processed by the NVD and our routine procedure (RP). Performances of the NVD were compared with RP. Samples only positive by the NVD assay were investigated by external PCR assays. Sensitivity and specificity (Se/Sp) and time from sample receipt to results were determined for each method. The NVD was also evaluated against 77 additional samples positive for a wide range of parasites. *Results*: Overall positivity rate was 16.9% for RP compared with 34% using the NVD assay, and 164 samples (66%) were negative by both methods. Only 30 positive samples (12%) showed full concordance between RP and NVD. Fifty-three discordant samples were sent for external investigations. Except for *Giardia intestinalis* and *Trichuris* spp., higher Se was observed for the NVD assay for *Blastocystis* spp. (100% *vs.* 63%), *Dientamoeba fragilis* (100% *vs.* 0%), *Schistosoma* spp. (100% *vs.* 17%), and *Enterobius vermicularis* (100% *vs.* 67%) but roughly similar to RP for the remaining parasites tested. False-positive results were identified for *Blastocystis* spp., *G. intestinalis*, and *Trichuris* spp. using the NVD assay. The NVD mostly provides a diagnosis on the day of sample receipt compared with a mean of three days with RP. *Conclusions*: Besides some limitations, the NVD is a new diagnostic strategy allowing rapid and high-plex detection of gastrointestinal parasites from unpreserved stools.

## Abstract (fr)

*Objectifs* : Nous présentons la première évaluation du kit Novodiag^®^ Stool parasite (NVD) marqué CE-IVD, permettant la détection rapide de 26 cibles distinctes dans les selles (protozoaires, helminthes et microsporidies). *Méthodes* : Un total de 254 échantillons (*n* = 205 patients) a été traité prospectivement par le NVD et notre procédure de routine (PR). Les performances du NVD ont été comparées à celles de la PR. Seuls les échantillons positifs au test NVD ont été étudiés par des PCR externes. La sensibilité et la spécificité (Se/Sp) ainsi que le temps écoulé entre la réception de l’échantillon et les résultats ont été déterminés pour chaque méthode. Le NVD a également été évalué par rapport à 77 échantillons supplémentaires positifs pour un large éventail de parasites. *Résultats* : Le taux de positivité global était de 16,9 % pour la PR contre 34 % avec le NVD, et 164 échantillons (66 %) étaient négatifs par les deux méthodes. Seuls 30 échantillons positifs (12 %) ont montré une concordance complète entre la PR et le NVD. Cinquante-trois échantillons discordants ont été envoyés pour des investigations externes. À l’exception de *Giardia intestinalis* et de *Trichuris* spp., des Se plus élevées ont été observées pour le test NVD pour *Blastocystis* spp. (100 % contre 63 %), *Dientamoeba fragilis* (100 % contre 0 %), *Schistosoma* spp. (100 % contre 17 %), *Enterobius vermicularis* (100 % contre 67 %) mais étaient à peu près similaires à la PR pour les autres parasites testés. Des faux positifs ont été identifiés pour *Blastocystis* spp., *G. intestinalis* et *Trichuris* spp. en utilisant le NVD. Le NVD fournit le plus souvent un diagnostic le jour de la réception du prélèvement contre une moyenne de trois jours avec la PR. *Conclusions* : Malgré quelques limites, le test NVD est une nouvelle stratégie de diagnostic permettant une détection rapide et high-plex des parasites gastro-intestinaux à partir de selles non conservées.

## Introduction

Besides bacteria and viruses, various parasites and microsporidia account for a significant part of gastrointestinal diseases worldwide [30]. Prevalence of these parasites varies greatly between countries, with higher prevalence usually being reported in tropical areas and mainly being related to poor sanitation and hygiene, and elevated contamination of water resources or soils. In high-resource countries, these diseases are dominated by protozoan parasites and microsporidia, although their respective prevalence is probably underestimated as they are thought to be underdiagnosed. Yet, in many countries, routine microscopy remains the gold standard for the diagnosis of intestinal parasite diseases. Besides the diagnosis of gastrointestinal diseases, parasite stool screening is also part of the general screening of stool donors in the context of fecal microbiota transplantation [5]. However, it exhibits several limitations like being resource-consuming and displaying limited performances [18]. In addition, it is usually recommended to process multiple samples to ensure sufficient sensitivity. In this context, it is not surprising that alternative approaches relying on antigen detection or DNA-based techniques have been developed, first in-house PCRs then commercial assays. A large range of commercial assays is now available on the market, including syndromic panels or addressing specifically the issue of gastrointestinal parasites, primarily protozoans. These assays have been the subject of many evaluation studies or reviews [20, 31].

The first commercially available multiplex PCR assay specifically developed for the detection of both helminths and microsporidia has recently been evaluated [3]. Combined with a previously developed assay targeting several protozoan species, once automated on a DNA extraction platform, it allows high-plex detection of 15 gastrointestinal pathogens [2, 3]. Despite this improvement, some major pathogens are not included in this panel, including *Schistosoma* spp., and therefore cannot be diagnosed. In addition, *Enterocytozoon* and *Encephalitozoon* species, could not be differentiated, illustrating the need for additional techniques to allow their identification as they require distinct therapeutic strategies.

Recently, a novel commercial and CE-IVD marked assay (Novodiag^®^ Stool Parasites, Mobidiag, Espoo, Finland), was approved. This technique relies on cutting-edge technology integrating DNA purification, PCR amplification and microarray hybridization/detection into a single, lab-on-chip cartridge. It allows for on-demand testing and high-plex detection of 26 distinct targets encompassing protozoans, helminths including nematodes and trematodes, and two microsporidian genera, from unpreserved stools. Thanks to fast processing of the samples, which includes mechanical disruption and ready-to-use cartridges, results are obtained within 90 min. Here we provide the first clinical evaluation of this new assay.

## Materials and methods

### Ethics

This study has been approved by our local ethic committee and registered under reference TS005-BIO.2019_6.

### Prospective study

**Figure 1:**
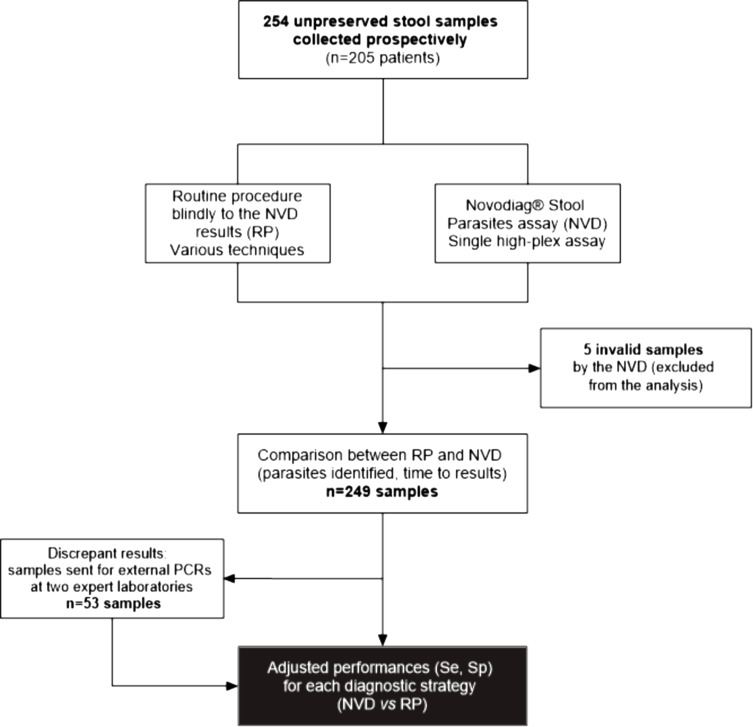
Study flow chart.

Two hundred and fifty-four unpreserved stool samples received at the Laboratory of Parasitology and Medical Mycology, Nantes University Hospital, France were processed using the Novodiag^®^ Stool Parasites assay (NVD), according to the manufacturer’s instructions (see Table S1 for the list of parasites included in the panel). In parallel, the same samples were simultaneously analyzed according to our routine procedures (RP, considered here the reference method), blindly to the results of the NVD assay (see Fig. 1). The routine procedure, which was performed by skilled microscopists, systematically included microscopic examination of the fresh unpreserved stool samples before and after a concentration step (Para-Selles^®^ Plus, Biosynex, Illkirch-Graffenstaden, France). Depending on the context (clinical symptoms, history of travel abroad, eosinophilia, etc.), additional techniques were performed: a further additional but different concentration step (Para-Selles^®^ Plus), modified Ziehl–Neelsen staining for coccidiosis, two species-specific in-house single plex real-time PCR assays targeting *Enterocytozoon bieneusi* and *Encephalitozoon intestinalis*/*E. hellem* [6], and agar plate culture according to Arakaki for strongyloidiasis [1]. Samples that were positive only by the NVD were subjected to previously published PCRs at two expert laboratories. These external investigations were conducted at Clermont-Ferrand University Hospital, France, for *Blastocystis* spp. and *D. fragilis* [25, 27] and at Elisabeth-TweeSteden Hospital, the Netherlands, for the remaining parasites [15]. Samples negative by the NVD but positive by RP were defined as false-negative samples of the NVD. Conversely, samples positive by the NVD assay but negative by both RP and the external assay were considered false-positive samples of the NVD. Finally, samples negative by RP but positive by the NVD assay and the external assay, were considered false-negative by RP. Sensitivity (Se) and specificity (Sp) were then determined for each diagnostic strategy. The time to complete sample analysis (interval between sample receipt at our laboratory and final results) was also collected for each sample and for each diagnostic strategy. Samples “invalid” by the NVD (i.e., internal control not detected) were excluded from the analysis. A flowchart of the design of the study is given in Figure 1.

### Retrospective study

In addition to the prospective evaluation study, 77 additional frozen stool samples, positive for a wide array of parasites and collected at our hospital and five other laboratories in France were also processed using the NVD assay. This additional set of samples included 21 distinct parasites, among which 14 were part of the diagnostic panel of the high-plex assay.

## Results

Overall, 249 (*n* = 201 patients) of the 254 stool samples (*n* = 205 patients) included in the prospective study, could be analyzed (5 invalid samples with the NVD, 2%). The positivity rate was 21.3% for RP (53 of 249 samples) but 16.9% (42 of 249) when excluding parasites not targeted by the NVD assay (i.e., non-pathogenic amoeba species and flagellates). By comparison, the positivity rate of the NVD was 34.1% (85 of 249). One hundred and sixty-four samples (65.9%) were negative by both diagnostic strategies.

**Figure 2:**
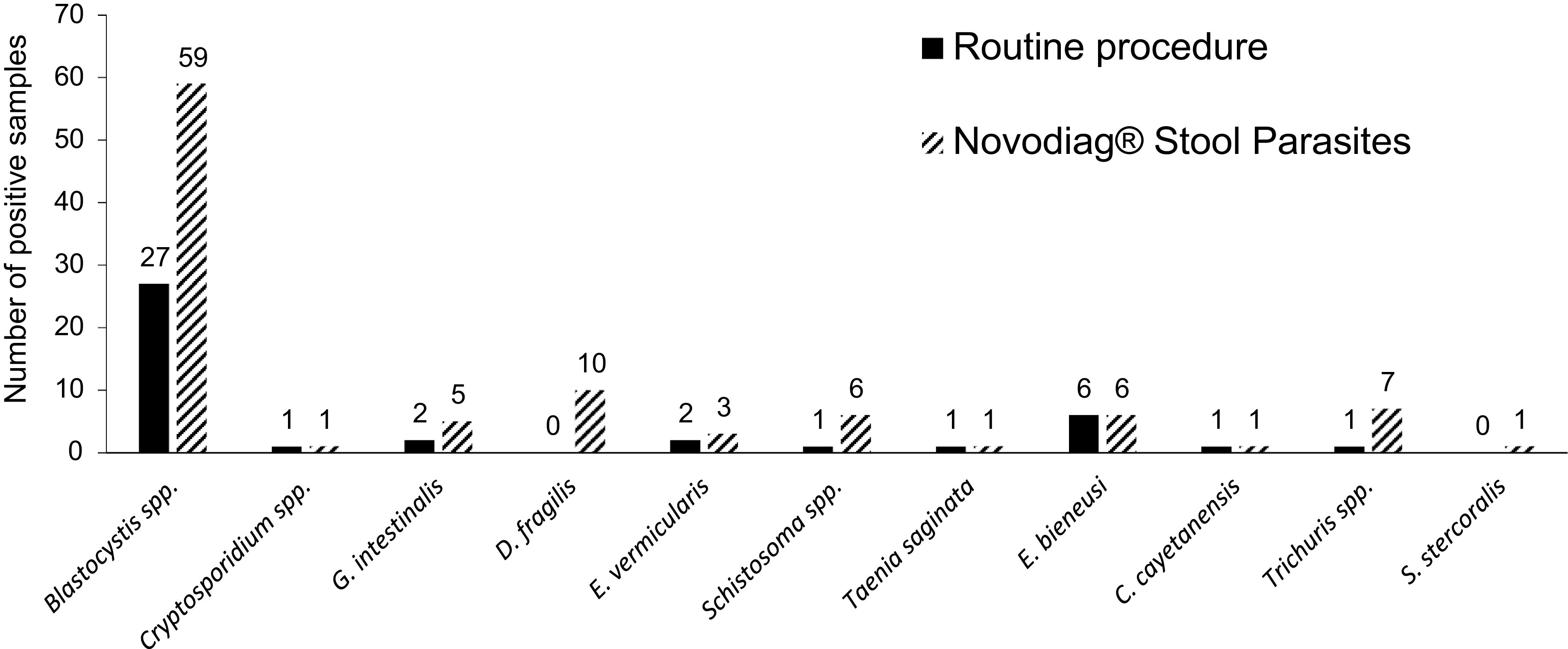
Diversity of the parasites identified in the prospective cohort according to each diagnostic strategy (*n* = 249 samples). *Note*: Additional parasites not included in the Novodiag^®^ panel were identified by microscopy: *Entamoeba coli* (*n* = 17), *Endolimax nana* (*n* = 18), and *Chilomastix mesnili* (*n* = 2).

Distribution of the 11 different parasites identified in this prospective cohort, according to each diagnostic strategy, is given in Figure 2. When positive, most samples displayed a single parasite (72 of 85, 84.7% for NVD, compared with 38 of 40, 95% for RP). Thirty samples (12%) showed full concordance between both diagnostic approaches (i.e., perfect match between the parasites identified by RP and NVD). Nine samples (3.6%) showed partial concordance (i.e., incomplete match between RP and NVD), whereas 49 (19.7%) showed no concordance. *Blastocystis* spp. ranked first as they were identified in 27 samples by RP (10.8%) compared with 59 samples using the NVD assay (23.7%). A higher detection rate with the NVD assay was also noted for other parasites: *Dientamoeba fragilis* was identified in 10 stool samples compared with none by RP, and six samples were positive for *Schistosoma* spp. using the NVD compared with a single positive sample using RP. Two of the positive samples for *Schistosoma* by the NVD assay but negative by microscopy were from patients recently diagnosed with schistosomiasis. The complete list of samples sent for external investigations to resolve the discrepancies is given in Table S2.

**Figure 3:**
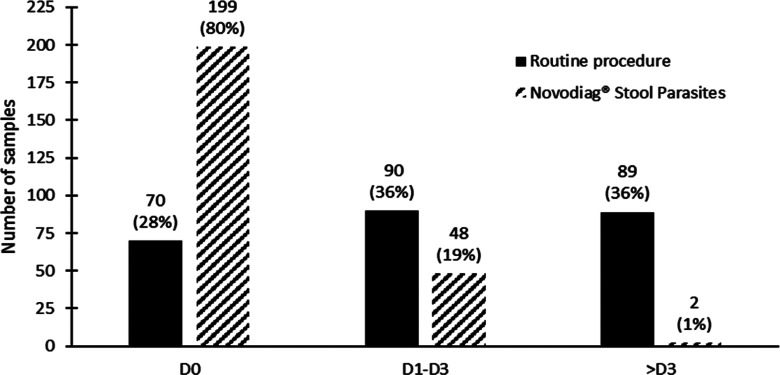
Time to final results according to each diagnostic strategy (routine procedure *vs*. Novodiag^®^ Stool Parasites) during the prospective study (*n* = 249 samples): D: day from sample arrival at the clinical laboratory to final results.

In-depth investigations of the discrepancies by external PCRs revealed that all samples negative by RP but positive by the NVD assay for *D. fragilis* (*n* = 10), *E. vermicularis* (*n* = 1) and *Schistosoma* (*n* = 3) were positive by the external PCR assays (i.e., false-negative by RP). External investigations also revealed that microscopy failed to detect some *Blastocystis* spp. (*n* = 16). In some instances, external PCRs could not confirm the findings of the NVD assay (Table S2). These samples considered false positives of the NVD were observed for *Blastocystis* spp. (*n* = 16), *G. intestinalis* (*n* = 4) and *Trichuris* spp. (*n* = 7). Lastly, adjusted analytical performances for the NVD and RP are given in Table 1. Except for *G. intestinalis* and *Trichuris* spp., sensitivity was higher for the NVD, for *Blastocystis* spp. (100% *vs.* 62.8%), *D. fragilis* (100% *vs.* 0%), *Schistosoma* spp. (100% *vs.* 16.7%), and *E. vermicularis* (100% *vs.* 66.7%). Identical performances for both diagnostic strategies were observed for *Cryptosporidium* spp., *Taenia saginata*, and *E. bieneusi*. The time to complete sample analysis evaluated during the prospective study is outlined in Figure 3. Although a mean of 3 days was required using RP to obtain the complete results, due to multiple techniques, the NVD assay mostly provided a final diagnosis on the day of sample receipt.

**Table 1:** Comparative performances between routine procedure and the Novodiag^®^ Stool Parasites assay on the prospective cohort (*n* = 249 samples).

Parasite of interest	Diagnostic strategy	Positive samples (*n*=)	Negative samples (*n*=)	False-positive samples* (*n*=)	False-negative samples* (*n*=)	Se (%)	Sp (%)
*Blastocystis* spp*.*	Routine	27	206	NA	16	62.8	NA
	Novodiag^®^	43	190	16	0	100	92.2
*Dientamoeba fragilis*	Routine	0	239	NA	10	0	NA
	Novodiag^®^	10	239	0	0	100	100
*Giardia intestinalis*	Routine	2	247	NA	0	100	NA
	Novodiag^®^	1	243	4	1	50	98.4
*Cryptosporidium* spp*.*	Routine	1	248	NA	0	100	NA
	Novodiag^®^	1	248	0	0	100	100
*Schistosoma* spp.^a^	Routine	1	243	NA	5	16.7	NA
	Novodiag^®^	6	243	0	0	100	100
*Taenia saginata*	Routine	1	248	NA	0	100	NA
	Novodiag^®^	1	248	0	0	100	100
*Enterobius vermicularis*	Routine	2	246	NA	1	66.7	NA
	Novodiag^®^	3	246	0	0	100	100
*Trichuris* spp*.*	Routine	1	248	NA	0	100	NA
	Novodiag^®^	0	241	7	1	0	97.2
*Enterocytozoon bieneusi*	Routine	6	243	NA	0	100	NA
	Novodiag^®^	6	243	0	0	100	100

NA: Not appropriate as routine procedure was considered the reference method (i.e., samples positive by RP were considered true positive).

* False-negative or false-positive were determined based on external PCR investigations.

a Except one identified to the genus level (*Schistosoma* spp.) all remaining *Schistosoma*-positive samples were identified as *S. mansoni* by the NVD and RP.

To challenge the NVD on a larger number of parasites, this technique was further evaluated on a collection of frozen positive stool samples. In all, 74 of 77 samples (three invalid samples) could be analyzed (Table 2). All samples known to be positive for *T. saginata* (*n* = 4), *Cystoisospora belli* (*n* = 2), *Entamoeba histolytica (*n = 2), *Ascaris lumbricoides* (*n* = 1), *Strongyloides stercoralis* (*n* = 4) and hookworms (*n* = 4) were also positive by the NVD (100% sensitivity/specificity). An almost perfect agreement was observed for *Cryptosporidium* spp., *G. intestinalis*, *E. vermicularis* and *E. bieneusi* with only a few false-negative results by the NVD assay (Table 2). Again, the NVD assay identified parasites which had been missed by microscopy: *Blastocystis* spp. (*n* = 10), *D. fragilis* (*n* = 2), and *S. mansoni* (*n* = 2). Both false-negative *S. mansoni* samples by RP were from two patients recently diagnosed with schistosomiasis. The NVD assay was negative for a sample positive with a single *S. mansoni* egg by microscopy, obtained from a patient diagnosed and treated for schistosomiasis in 2017. Only four out of the 8 positive samples for *Trichuris* spp. were detected by the NVD, confirming its poor performance for this target. Finally, no cross-reactivity was evidenced with parasites not included in the high-plex panel (Table 2).

**Table 2:** Performances of the Novodiag^®^ Stool Parasites assay on a collection of positive samples (*n* = 74 samples, retrospective study).

Parasite of interest	Positive samples according to routine procedure (*n*=)	Positive samples by the Novodiag^®^ assay (*n*=)
*Blastocystis* spp*.*	16	26
*Dientamoeba fragilis*	0	2
*Giardia intestinalis*	14	12
*Cryptosporidium* spp*.*	8	7
*Cystoisospora belli*	2	2
*Entamoeba histolytica*	2	2
*Schistosoma mansoni*	9	10
*Taenia* spp*.*	4	4^a^
*Ascaris lumbricoides/suum*	1	1
*Enterobius vermicularis*	3	2
*Strongyloides stercoralis*	4	4
Hookworms	4	4^b^
*Trichuris* spp*.*	8	4
*Enterocytozoon bieneusi*	8	7
Other parasites*	42	0

a All identified as *T. saginata/Taenia asiatica* by the Novodiag^®^ assay.

b Identified as *N. americanus* (*n* = 3), *A. duodenale* (*n* = 1) by the Novodiag assay^®^.

* Other parasites not targeted by the Novodiag^®^ assay included: *Dicrocoelium dendriticum* (*n* = 1), *Entamoeba coli* (*n* = 16), *Endolimax nana* (*n* = 12), *Entamoeba dispar* (*n* = 6), *Entamoeba hartmanni* (*n* = 3), *Iodamoeba butschlii* (*n* = 1) and unidentified flagellates (*n* = 4).

## Discussion

The present study reports the first evaluation of the NVD assay compared with routine procedures, in real-life conditions. One strength of the prospective study was to include specific external PCR assays in order to resolve the discrepant results, a strategy which is often missing in similar studies but offers better evaluation of assay performances [3, 28, 29]. Most discrepancies were observed with *Blastocystis* spp., *D. fragilis* and *S. mansoni*, for which detection rates were higher using the NVD assay. Of note, the lack of *D. fragilis* detection by microscopy was expected as RP was performed on unpreserved stools and trichrome permanent staining is not performed in our laboratory. Except for *Blastocystis* spp., for which the improved detection rate also unmasked false-positive samples, the higher sensitivity of the NVD for *D. fragilis* and *S. mansoni* is in agreement with previous studies using PCR assays [3, 10, 13, 21, 25]. Another strength was to challenge the NVD assay on a collection of positive samples. This allowed us to consolidate our findings for *Cryptosporidium* spp., *E. bieneusi* and *T. saginata*, which were poorly represented in the prospective study*.* Likewise, it provides additional data for soil-transmitted helminths, *E. histolytica and C. belli*, for which the NVD assay performed well although the number of positive samples remains low. On the contrary, this set of samples confirmed the poor performance of the NVD for *Trichuris* spp., although the sample processing with the NVD includes a bead-beating step that is known to improve the detection of *Trichuris* spp. [4, 14]. The reduced performances of the NVD for *G. intestinalis*, observed during the prospective study, were unexpected as there is an overall consensus about the superiority of DNA detection over microscopy for this protozoa [2, 17, 21, 24]. Of note, better performances were observed during the retrospective study which included a higher number of *Giardia*-positive samples. These weak performances for the detection of *Trichuris* spp. and to a lesser extent *G. intestinalis* which are already known by the manufacturer (outlined in the technical note of the NVD assay), require specific optimizations.

Besides these limitations, the main advantages of this new assay include: (i) a high-plex format, allowing the detection of the 26 most common protozoa, helminths and microsporidia in a single run; (ii) a fast turnaround time allowing results within 90 min with few invalid results; (iii) higher performances for the detection of *S. mansoni* which is of major interest considering the notoriously poor sensitivity of microscopy [18]; (iv) its ability to provide species identification which can be useful for epidemiological purposes, as illustrated with hookworms and *Taenia* species eggs which cannot be identified to the species level using microscopy [9]. Here, except one identified as *Ancylostoma duodenale*, all hookworm-positive samples were *Necator americanus*. Further investigations are required to assess the ability of the NVD to identify the emerging species *Ancylostoma ceylanicum* [8]. The NVD assay also allows for the screening of underdiagnosed pathogens such as microsporidia and *Cryptosporidium* spp., which are often overlooked by physicians as their diagnosis requires specific techniques. Furthermore, its ability to discriminate *E. bieneusi* from *Encephalitozoon* species is of specific interest as distinct treatment regimens are required to treat these opportunistic fungi [11].

Because of the intermittent shedding of parasites, notably helminths eggs or larvae and the overall low sensitivity of microscopic methods, it is usually recommended to repeat parasite stool examination on different days, to ensure high sensitivity [31]. This strategy is controversial in populations with a low prevalence of the disease, as discussed elsewhere [7]. An alternative and cost-effective option is to limit the examination of a second specimen only when the first sample is negative and in symptomatic patients, with a third specimen for patients remaining negative despite a high index suspicion [23]. Our study was not designed to answer whether a single stool PCR performed similarly to RP on three consecutive samples, because only a few patients in our study had multiple samples available for analysis. Finally, a shortage of NVD reagents due to the COVID-19 pandemic, led us to stop this evaluation at some points of the prospective study.

Not all genera/species included in the panel of the NVD assay could be tested, which is a limitation of the present study. However, these missing parasites are the rarest identified in stool samples in European countries, which underlines the difficulty to assess the performances of multiplex panels in countries with a low prevalence of these diseases [12, 16, 19, 21, 22, 26].

## Conclusion

To conclude, one major advantage of this high-plex assay is the rapid turnaround time which is explained by a reduced hands-on time together with the capability of the instrument to process four samples simultaneously, which makes it possible to reduce diagnostic delays. Importantly, although time to results is largely dependent on each laboratory organization and diagnostic strategies, compared with RP which requires multiple concentrations, staining methods as well as additional techniques in some cases, the NVD is simpler and therefore faster, providing a result within 90 min. The fully automated Novodiag^®^ Stool Parasites assay is an alternative approach to labor-intensive microscopy-based methods especially in settings or countries with low parasite prevalence or personnel with reduced skills in parasitological diagnosis. Besides the diagnosis of gastrointestinal diseases, this technique also represents an attractive approach for rapid donor stool screening in the context of fecal microbiota transplantation [5]. This study provides better knowledge of the performances of the NVD in real-life conditions, which we hope will be of great help to determine its utility in the diagnostic algorithm of gastrointestinal parasites.

## Conflict of interest

The authors declared no conflict of interest.

## Funding

This study was supported by internal funding from Nantes University Hospital.

## Supplementary material

Supplementary material is available at https://www.parasite-journal.org/10.1051/parasite/2022026/olm. *Table S1*. High-plex panel of the Novodiag^®^ Stool Parasites assay. *Table S2*. Results of external investigations for samples positive by Novodiag^®^ assay and negative by the routine procedure (*n* = 53).
