# Supplementary material for: The Novodiag® Stool parasites assay, an innovative high-plex technique for fast detection of protozoa, helminths and microsporidia in stool samples: a retrospective and prospective study
Source: Parasite. 2022 May 13;29:27. doi: 10.1051/parasite/2022026 (PMC9102957; doi:10.1051/parasite/2022026)
Supplement: Supplementary file 1 — Table S1. High-plex panel of the Novodiag® Stool Parasites assay. [file parasite-29-27-s1.pdf]

Supplementary data

Table S1 High-plex panel of the Novodiag® Stool Parasites assay

**Genus/species targeted by the Novodiag® Stool Parasites**
Ancylostoma duodenale
Ascaris lumbricoides / suum [a]
*Balantidium coli**
Blastocystis spp.
Clonorchis sinensis / Opisthorchis spp. / *Metorchis* spp . [a*]
Cryptosporidium spp. [b]
Cyclospora cayetanensis
Cystoisospora belli
Dientamoeba fragilis
Diphyllobothrium latum / nihonkaiense [a*]
Encephalitozoon spp. [c*]
Entamoeba histolytica
Enterobius vermicularis
Enterocytozoon bieneusi
Fasciola spp. [d*]
*Fasciolopsis buski**
Giardia intestinalis
*Hymenolepis diminuta**
*Hymenolepis nana**
Necator americanus
Schistosoma mansoni
Schistosoma spp. [e]
Strongyloides stercoralis
Taenia saginata / asiatica [a]
*Taenia solium**
Trichuris spp.

[a] These species cannot be differentiated
[b] At least these species can be detected *C. hominis, C. meleagridis, C. parvum, C. ubiquitum* and *Cryptosporidium* spp. *Chipmunk genotype I.*
[c] At least *E. cuniculi, E. hellem and E. intestinalis* can be detected.
[d] *F. hepatica* and *F. gigantica* can be detected.
[e] At least *S. bovis, S. curassoni, S. edwardiense, S. haematobium, S. hippopotami, S. incognitum, S. intercalatum, S. japonicum, S. leiperi, S. malayensis, S. mansoni, S. margrebowiei, S. mattheei, S. mekongi and S. sinensium* can be detected.
*No positive sample for this species could be included in the present study.
