# Supplementary material for: The Novodiag® Stool parasites assay, an innovative high-plex technique for fast detection of protozoa, helminths and microsporidia in stool samples: a retrospective and prospective study
Source: Parasite. 2022 May 13;29:27. doi: 10.1051/parasite/2022026 (PMC9102957; doi:10.1051/parasite/2022026)
Supplement: Supplementary file 2 — Table S2. Results of external investigations for samples positive by Novodiag® assay and negative by the routine procedure (n = 53). [file parasite-29-27-s2.pdf]

<u>Table S2</u> Results of external investigations for samples positive by Novodiag® assay and negative by the routine procedure (n=53)

Note: only parasites included in the Novodiag® panel and showing discrepant results between routine procedure and the Novodiag® assay are indicated in this table.

Sample No.	Routine procedure	Novodiag® Assay	Results of External PCR assays	Final status
P1	-	*Blastocystis* sp.	*Blastocystis*-positive	FN microscopy
P2	-	*Blastocystis* sp.	*Blastocystis*-positive	FN microscopy
J1	-	*G. intestinalis*	*Giardia*-negative	FP Novodiag®
J2	-	*E. vermicularis*	*Enterobius*-positive	FN microscopy
P3	-	*Blastocystis* sp.	*Blastocystis*-positive	FN microscopy
P4	-	*Blastocystis* sp.	*Blastocystis*-positive	FN microscopy
P5	-	*Blastocystis* sp.	*Blastocystis*-positive	FN microscopy
P6	-	*Blastocystis* sp.	*Blastocystis*-negative	FP Novodiag®
P7	-	*D. fragilis*	*D. fragilis*-positive	FN microscopy
P8	-	*Blastocystis* sp.<br>*D. fragilis*	*Blastocystis*-negative<br>*D. fragilis*-positive	FP Novodiag®<br>FN microscopy
P9	-	*Blastocystis* sp.<br>*D. fragilis*	*Blastocystis*-positive<br>*D. fragilis*-positive	FN microscopy
P10	*C. cayetanensis* [a]	*C. cayetanensis*<br>*D. fragilis*	*Cyclospora*-negative<br>*D. fragilis*-positive	Undetermined<br>FN microscopy
P11		*Blastocystis* sp.	*Blastocystis*-positive	FN microscopy
P12	-	*Blastocystis* sp.	*Blastocystis*-negative	FP Novodiag®
P13	-	*Blastocystis* sp.	*Blastocystis*-positive	FN microscopy
P14	-	*Blastocystis* sp.<br>*D. fragilis*	*Blastocystis*-negative<br>*D. fragilis*-positive	FP Novodiag®<br>FN microscopy
J6	-	*G. intestinalis*	*Giardia*-negative	FP Novodiag®
P15	-	*Blastocystis* sp.	*Blastocystis*-positive	FN microscopy
P16	-	*Blastocystis* sp.	*Blastocystis*-negative	FP Novodiag®
P17	-	*Blastocystis* sp.	*Blastocystis*-positive	FN microscopy
J7	-	*Trichuris* sp.	*Trichuris*-negative	FP Novodiag®
P18	-	*Blastocystis* sp.	*Blastocystis*-negative	FP Novodiag®
P19	-	*Blastocystis* sp.	*Blastocystis*-positive	FN microscopy
P20	-	*Blastocystis* sp.	*Blastocystis*-negative	FP Novodiag®
J8	-	*Trichuris* sp.	*Trichuris*-negative	FP Novodiag®
J9	-	*Trichuris* sp.	*Trichuris*-negative	FP Novodiag®
P21	-	*Blastocystis* sp.	*Blastocystis*-positive	FN microscopy
P22	-	*Blastocystis* sp.	*Blastocystis*-negative	FP Novodiag®
J10	-	*Giardia intestinalis*	*Giardia*-negative	FP Novodiag®
P23	-	*D. fragilis*	*D. fragilis*-positive	FN microscopy
P24	-	*Blastocystis* sp.	*Blastocystis*-negative	FP Novodiag®
J11	-	*Trichuris* sp.	*Trichuris*-negative	FP Novodiag®
P25	-	*Blastocystis* sp.	*Blastocystis*-negative	FP Novodiag®
P26	-	*Blastocystis* sp.	*Blastocystis*-negative	FP Novodiag®
P27	-	*D. fragilis*	*D. fragilis*-positive	FN microscopy
P28	-	*D. fragilis*	*D. fragilis*-positive	FN microscopy
J12	-[b]	*S. mansoni*<br>*S. stercoralis*	Schistosoma-positive<br>Strongyloides-negative	FN microscopy<br>Undetermined
P29	-	*Blastocystis* sp.	*Blastocystis*-negative	FP Novodiag®
P30	-	*Blastocystis* sp.<br>*D. fragilis*	*Blastocystis*-positive<br>*D. fragilis*-positive	FN microscopy
P31	-	*D. fragilis*	*D. fragilis*-positive	FN microscopy
P32	-	*Blastocystis* sp.	*Blastocystis*-negative	FP Novodiag®
J13	-	*S. mansoni*	Schistosoma-positive	FN microscopy
J14	-	*Trichuris* sp.	*Trichuris*-negative	FP Novodiag®
J15	-	*Trichuris* sp.	*Trichuris*-negative	FP Novodiag®

P33	-	*Blastocystis* sp.	*Blastocystis*-positive	FN microscopy
P34	-	*Blastocystis* sp.	*Blastocystis*-negative	FP Novodiag®
P35	-	*Blastocystis* sp.	*Blastocystis*-negative	FP Novodiag®
P36	-	*Blastocystis* sp.	*Blastocystis*-negative	FP Novodiag®
J16	-	*S. mansoni*	Schistosoma-positive	FN microscopy
P37	-	*Blastocystis* sp.	*Blastocystis*-positive	FN microscopy
P38	-	*Blastocystis* sp.	*Blastocystis*-positive	FN microscopy
J17	-	*Trichuris* sp.	*Trichuris*-negative	FP Novodiag®
J18	-	*G. intestinalis*	*Giardia*-negative	FP Novodiag®

Footnotes: FN: False-negative; FP: False-positive

[a] Microscopic examination was doubtful (a single oocyst was evidenced). This sample, being negative by the external *C. cayetanensis*-specific PCR assay was therefore classified as "uncertain".

[b] This patient had positive serology for both strongyloidiasis and schistosomiasis. As microscopy was negative as well as the external PCR assay for *S. stercoralis*¸ possible cross-reactivity between serological tests cannot be excluded. The sample was therefore classified as "uncertain".
